# Sustained pain relief from radiofrequency ablation of the superior cluneal nerves using a bipolar palisade technique: A case report

**DOI:** 10.1016/j.inpm.2024.100425

**Published:** 2024-07-16

**Authors:** Theodore Cohen

**Affiliations:** a Department of Anesthesiology, University of Miami, Miami, FL, USA; b Don Soffer Clinical Research Center, 1120 N.W. 14th Street, Suite 937, Miami, FL, 33136, USA

## Abstract

Previous authors have described the anatomy of the superior cluneal nerves with medial, intermediate, and lateral branches as they pass over the iliac crest. Prior authors describe a technique for radiofrequency of the superior cluneal nerves with needle placement walking off of the superior border of the iliac crest with needle redirection by sensory testing and a monopolar radiofrequency lesion. This is a case report of a patient with sustained pain relief after performing a radiofrequency ablation of the superior cluneal nerves utilizing a bipolar palisade technique.

## 1 Introduction

The superior cluneal nerves are the cutaneous branches of the dorsal rami of T11-L5 that pierce the thoracolumbar fascia superior to the iliac crest [1]. The branches of the superior cluneal nerves are the medial, intermediate, and lateral branches. Proposed mechanisms of pain from the superior cluneal nerves are entrapment as it passes through the thoracolumbar fascia caudal to the iliac crest through a fibrous-osseous tunnel or iatrogenic injury by bone graft harvesting at iliac crest [1]. On physical exam patients with superior cluneal neuropathy can have tenderness at the superior iliac crest approximately 7 cm from midline, or pain with extension, lateral flexion or rotation [1]. The diagnosis of pain originating from the superior cluneal nerves can be confirmed with a diagnostic nerve block. If pain persists, more prolonged relief could be achieved by ablating the superior cluneal nerves. The prior published technique for superior cluneal nerve ablation from Visnjevac et al. [2] requires walking off the iliac crest and directing the RFA needle by responses to sensory testing.

## 2 Case description

The patient was a 75-year-old male presenting with approximately 3 years of positional pain in the right gluteal area. The patient had transient relief from previous right sacroiliac joint steroid injections by a previous provider and only 20–30 % relief from a right lateral branch radiofrequency ablation targeting his right sacroiliac joint. He continued to report positional right sided gluteal pain with standing and walking and a decision was made to target the patient's right superior cluneal nerves. The patient provided informed consent prior to all procedures performed. The patient had two fluoroscopic guided blocks of the superior cluneal nerves using the technique described by Gautam et al. [3] targeting the superior iliac crest approximately 7–8 cm lateral to midline at the L5 level. When the needle was in position at the iliac crest 0.5 ml of Omnipaque 240 was injected to confirm spread along the Iliac crest without vascular uptake on repeat fluoroscopic imaging, followed by injection of an 8 cc solution of 7 cc of 0.25 % bupivacaine and 40 mg of methylprednisolone. The volume of the injectate and use of steroid is described by Waldman [4] for superior cluneal nerve block with a landmark technique. Steroid was added to potentially prolong the block and add a therapeutic response in the setting of the patient's pain being positional. The patient reported that the pain returned to baseline approximately 5 weeks after the second block. We proceeded to a right radiofrequency ablation of the superior cluneal nerves using a bipolar palisade technique.

**Fig. 1:**
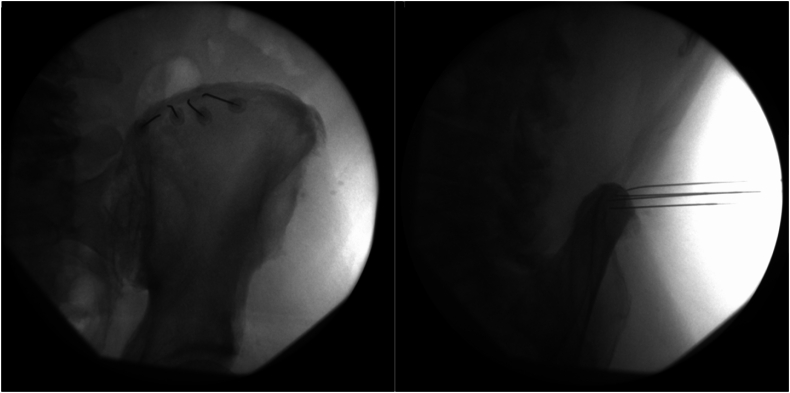
A(left) is posterior-anterior view of needle placement in palisade arrangement at superior border of iliac crest. Fig. 1B (right) is contralateral oblique view of the same needles at iliac crest.

The target site identified for the ablation was the same as the diagnostic blocks at the right iliac crest approximately 7–8 cm lateral to mid-line of lumbar 5 where the branches of the superior cluneal nerves (medial, intermediate and lateral) cross over the iliac crest. A total of four 100mm 20 gauge insulated curved radiofrequency needles with 10 mm active tips were placed at the superior edge of the iliac crest in the location previously described and in a palisade arrangement ≤ 1 cm apart (Fig. 1A). A contralateral oblique view was used to confirm depth relative to the iliac crest (Fig. 1B). Then bipolar sensory stimulation at 50 Hz 0.5 V was tested, patient reported pain at his right buttock in the distribution of his typical pain, motor stimulation at 2 Hz and 3V was tested and negative at each needle. Then the radiofrequency ablation probes were threaded through the needles and bipolar ablation was performed at 80 °C for 90 seconds through all the needles. His maximum NRS pain score at the follow up visit prior to the ablation was 10/10 and occurred with standing for 10–15 minutes. At the 4 week post ablation follow up visit he reported 60 % relief, he was able to stand for 30–45 minutes prior to experiencing more severe pain, and the maximum NRS pain score was 8/10. The discordancy of percentage relief and maximum NRS pain score reported was attributed to post ablation discomfort. At the 6-month post ablation follow up visit the patient reported ongoing 80–90 % relief with a maximum NRS pain score of 2/10. This high amplitude relief was sustained at the 12-month follow-up with a maximum NRS pain score reported at 3/10.

## 3 Discussion

The current published technique for superior cluneal nerve ablation from Visnjevac et al. [2] describes an approach where the RFA cannula is directed towards the superior border of the iliac crest approximately 7–8 cm from midline at the L5 level. The needle is then walked off the iliac crest superiorly with redirection of the needle determined by sensory testing with the desired result of concordant pain. When the concordant pain is achieved there is then a monopolar thermal lesion. Visnjevac discusses that the repositioning of the needle was typically lateral and, in some cases, medial repositioning reproduced the patient's symptoms. The author indicates that when using this technique it is unclear which branch is being ablated. While there were no serious adverse events reported for the 46 patients in this retrospective chart review, there is a potential risk for visceral penetration given that the needle was walked off the osseous landmark of the iliac crest.

A palisade is a fence of stakes. The term was used by Cosman [5] to describe a technique for RFA needle placement targeting the lateral branches for sacroiliac joint pain. The RFA needles (or cannulae) are placed in a straight row for the purpose of generating bipolar lesions between needles for a continuous palisade lesion.

The technique described in this case report has two main advantages over the technique described by Visnjevac et al. First, this is a technique that does not require walking off the iliac crest. For this case the needles were placed at the edge of the iliac crest in Palisade arrangement. By placing the needles on an osseous structure there is an inherent reduced risk of visceral penetration. Second, when performing this procedure, the specific branch of the superior cluneal nerves that is the source of the pain is unknown. By performing a strip lesion ablation using the technique described above there is a higher probability of capturing all 3 branches of the superior cluneal nerves. Sensory stimulation was used in this case, however this was not for needle repositioning but to confirm concordant pain.

Additionally, contralateral oblique imaging was performed of the iliac crest (Fig. 1B). Given the slope and shape of the iliac crest, lateral imaging would not provide adequate information of needle depth relative to the iliac crest. While this case used the superior iliac crest as an end point for needle placement minimizing the risk of advancing the needle too far into viscera, this image provides additional information confirming proper needle placement. Additionally, this view could be helpful in determining relative depth if performing the procedure as described by Visnjevac et al.

Regarding this patient's particular case, he reported a wide pain range as his pain was positional and his response to treatment was determined by maximum NRS pain scores and percentage relief that he reported. His limited NRS score response at the 4 week follow up was likely post ablation discomfort given the size of the lesion produced with the palisade technique. However, the patient was reporting this pain for approximately 3 years prior to this ablation and had sustained high amplitude responses 6 and 12 months post ablation. One limitation of this case is that the patient may have had a small component of pain from his right sacroiliac joint which was treated prior to the superior cluneal nerve ablation. There is also the limitation that this is a single case report.

## Funding

None.

## Declaration of competing interest

The authors declare the following financial interests/personal relationships which may be considered as potential competing interests:

Theodore Cohen reports administrative support, article publishing charges, and equipment, drugs, or supplies were provided by 10.13039/100006686University of Miami Miller School of Medicine. Theodore Cohen reports a relationship with University of Miami Miller School of Medicine that includes: employment. If there are other authors, they declare that they have no known competing financial interests or personal relationships that could have appeared to influence the work reported in this paper.
